# Integrated graphene oxide resistive element in tunable RF filters

**DOI:** 10.1038/s41598-020-70041-x

**Published:** 2020-08-04

**Authors:** Heba Abunahla, Rida Gadhafi, Baker Mohammad, Anas Alazzam, Mamady Kebe, Mihai Sanduleanu

**Affiliations:** 1 System-on-Chip Center, Electrical Engineering and Computer Science Department, Khalifa University of Science and Technology, Abu Dhabi, United Arab Emirates; 2 System-on-Chip Center, Mechanical Engineering Department, Khalifa University of Science and Technology, Abu Dhabi, United Arab Emirates; 3 Present Address: Department of Electrical Engineering, University of Dubai, Dubai, United Arab Emirates

**Keywords:** Electrical and electronic engineering, Electrochemistry

## Abstract

Adaptable communication systems are of great interest as they provide dynamic front end to accommodate the tunable spectrum management in advanced wireless systems. Memristor (acronym of memory resistor) is an emerging technology part of resistive RAM (RRAM) that has good potential for application in reconfigurable RF devices. The potentiality of using resistive switches for frequency tuning of high frequency RF filters is successfully explored in this article for the first time. Tunable RF filter is designed with detailed simulation using Ansys HFSS, and then correlated with measured results from experiment. As a proof of concept, a prototype of the tunable RF filter is fabricated by using a graphene oxide (GO) integrated with a conventional microstrip open stub notch filter. The resistor switching ability of the device is exploited for the frequency tuning. The resonating length of the notch filter is varied by changing the resistance of the active GO material between ‘HIGH’ (OFF) and ‘LOW’ (ON) resistance states. The measured results demonstrate the great potential of using RRAM in tunable RF devices. It also proves the possibility of tuning RF devices without any localized surface mount device (SMD) element or complex realization technique.

## Introduction

**Figure 1:**
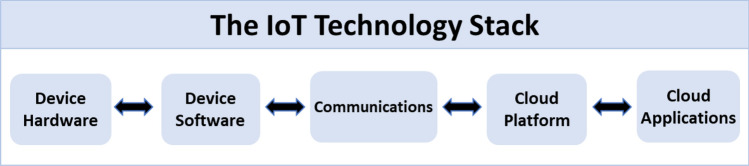
Basic components of the Internet of Things (IoT) stack.

Being a crucial component in radio frequency (RF) transceivers, filters play a major role in wireless communication, which is among the main pillars of IoT technology stack (Fig. 1)^1^. Over the last decades, the extensive use of wireless devices causes a huge increase in the amount of network participants and services^2^. The efficient use of radio spectrum can rectify this problem to some extent. Tunable filters are key components in dynamic spectrum management concepts like software defined radios and cognitive radios^3^. Such devices provide ideal filtering solution for communications, medical, defense, and other essential fields^4^. Tunable RF filters are being explored in the literature for many years^2,5–9^. Most of them are based on placing a localized SMD element, most commonly with a varactor^2,5,7–9^.

The memristor (MR) device is an emerging RRAM technology that was postulated by Chua in 1971^10^, and realized by HP lab in 2008^11^. MR device resistance can be changed in a nonvolatile way from one state (ON) to another (OFF) or vice versa. Such device has the potential to be deployed in many emerging applications such as neuromorphic, computing, memory, security and sensing^12–18^. Recently, RRAM switching ability has attracted researchers’ attention to be utilized in reconfigurable RF devices. Due to its superiority as a non-volatile technology leads to low energy consumption, RRAM device can potentially replace the conventional RF switches^18^. Comparing RRAM-based tunable RF components to PIN diode, RRAM has the advantage of being non-volatile. Thus, a one-time voltage bias should be applied to set the device to ON or OFF state, and then the device keeps remembering its last state even after disconnecting the bias voltage. On the other hand, the PIN diode is an active device, therefore requires a continuous bias voltage application in order to maintain the ON or OFF state^29,30^. Hence, RRAM technology is considered power efficient solution for tunable RF components. As for RF MEMS switching devices, they suffer from mechanical stress of the deposited metal layers^31^ which are brittle, reducing their reliability in wireless communications applications. Hence, the RRAM-based tunable RF component can stand longer periods of operation once the switching state is set.

Although many researchers have explored the possibility of integrating RRAM in reconfigurable RF circuits^18–28^, most of these findings are based on either simulation or numerical modeling^19–22^. Few attempts report fabricated RRAM to be used for RF circuitry,however, all of these attempts have used RRAM as standalone component to tune the resistance. On contrary, our approach is to integrate the planer RRAM with the metallization of the RF component. For example, the works provided in^23,24^ propose connecting the fabricated discrete RRAM as a variable resistor with other circuit components to demonstrate tunable filter behavior. The work provided in^25^ proposes deploying non-volatile resistive switches in phased antenna arrays. Although detailed fabrication and characterization are provided for the used resistive switches, the integration of these devices with phased antenna arrays is done only in simulations and the practical implementation and realization of the tunable RF device is missing. Thus, all the aforementioned state of the art does not provide a realization of stand-alone tunable filter integrated with planar RRAM.

This paper proves experimentally, for the first time, the possibility of using RRAM devices in planar RF filters that can operate at high frequencies. The switching ability of graphene oxide (GO)^32–34^ is utilized in fabricating a tunable standard open stub notch filter. The obtained results confirm the great potential of using resistive switching materials as tunable element in RF devices. Also, the measurements provided in this work confirmed the possibility of tuning RF devices without any localized surface mount device (SMD) element or complex realization technique. The challenges associated with integrating oxide material with the conductive material of the filter is also highlighted. This paper also presents a detailed simulation study of RRAM-based filters, which also agrees with our postulates and is used to guide the fabrication process. The potential results presented in this work open a trending area for RF researchers to explore the possibility of replacing conventional RF switches with RRAM switches.

The rest of the paper is organized as follows. “Experimental procedure” details the experimental procedures and the device structure. “Results and discussion” describes the simulation study of RRAM-based tunable notch filter. Also, planar GO-based RRAM devices that are suitable for the tunable filter application are presented, followed by the measurement results of the fabricated tunable filter. Finally, the work conclusions are provided in “Conclusion”.

## Experimental procedure

### Device fabrication

**Figure 2:**
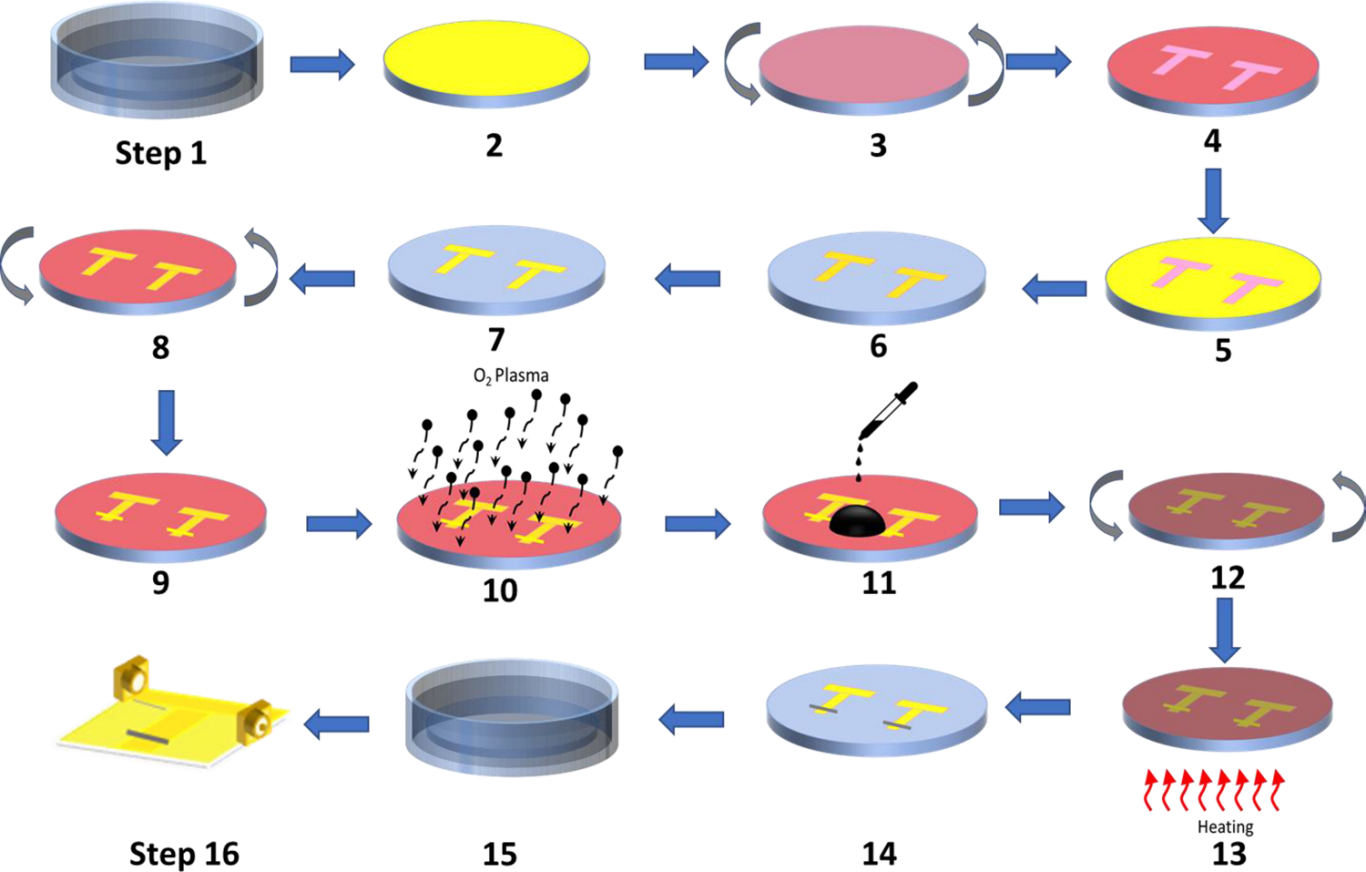
Steps of the fabrication procedure followed to fabricate the filter presented in this paper.

**Figure 3:**
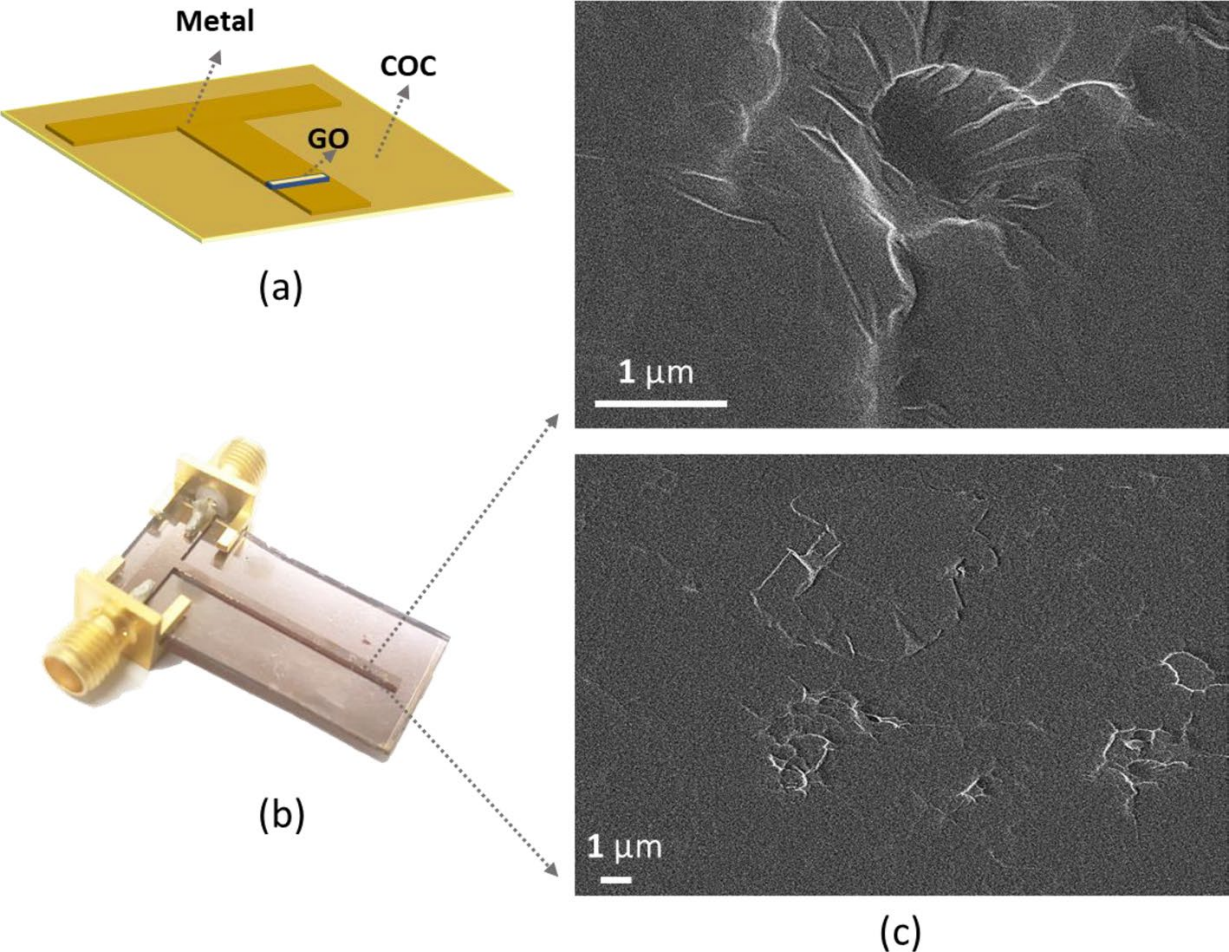
**(a)** Device schematic to show the planar RRAM-based filter structure. **(b)** A photo of the fabricated filter on Cyclic Olefin Copolymer (COC) substrate. **(c)** Scanning electron microphotograph of the deposited GO layer, top view, under secondary electron mode.

The tunable filter presented in this work is fabricated on Cyclic Olefin Copolymer (COC) wafer using standard microfabrication techniques. This substrate has been chosen as it provides excellent adhesion with GO^35^. The fabrication steps of RRAM-based tunable filter are shown in Fig. 2. In step 1, the COC wafer is cleaned by sonicating it in acetone, followed by isopropanol, and DI water baths. A metal deposition (gold for this work) is performed using sputter coating system as shown in step 2. The lithography process is then achieved by spin coating of thin layer of positive photoresist on top of the metal film (step 3). The photoresist layer is patterned using a photolithography system and then developed using a proper developer (steps 4 and 5). After that, the exposed metal layer is etched away using the associated metal etchant (steps 6). Then, the wafer is dipped in acetone to remove the photoresist layer (step 7). In steps 8–14, GO layer is deposited and patterned on the metal film using Plasma-enhanced lift-off procedure^35^. To elaborate, spin coating is used to deposit a thin layer of positive photoresist on the fabricated filter (step 8). This layer is then patterned and developed in step 9. After that, a layer of GO is deposited using a spin coater (steps 11 and 12), baking the wafer on a hot plate (step 13), and then removing the photoresist layer by acetone (step 14). Prior the deposition of GO, the device surface is treated by plasma for few minutes (step 10) to improve the adhesion between the GO and the surface. In step 15, the back side of the wafer is coated by the same metal used for filter fabrication. The final step is to attach the RF connectors to the fabricated filters. A schematic and photo of the fabricated filter are presented in Fig. 3a,b.

### Device characterization

The S-parameter of the fabricated filter is measured by the vector network analyzer. SOLT (Short, Open, Load, Thru) calibration is performed before S-parameter measurements. The measurements are taken when the RRAM device is OFF and after switching the device to ON state. The electrical characterization is performed using Keithley 4200-SCS Parameter Analyzer with pulse mode to screen for the RRAM devices functionality.

Microscopic images for the patterned GO film are obtained using scanning electron microscopy (SEM). In line with the work provided in^35^, Fig. 3c shows the GO film deposited in the filter gap. GO material has great potential to be deployed in flexible RRAM devices. Systematic study on the switching mechanism associated to GO-based RRAM is reported in^40^. In this paper, we utilize the switching ability of GO material to design and fabricate tunable notch filter.

**Figure 4:**
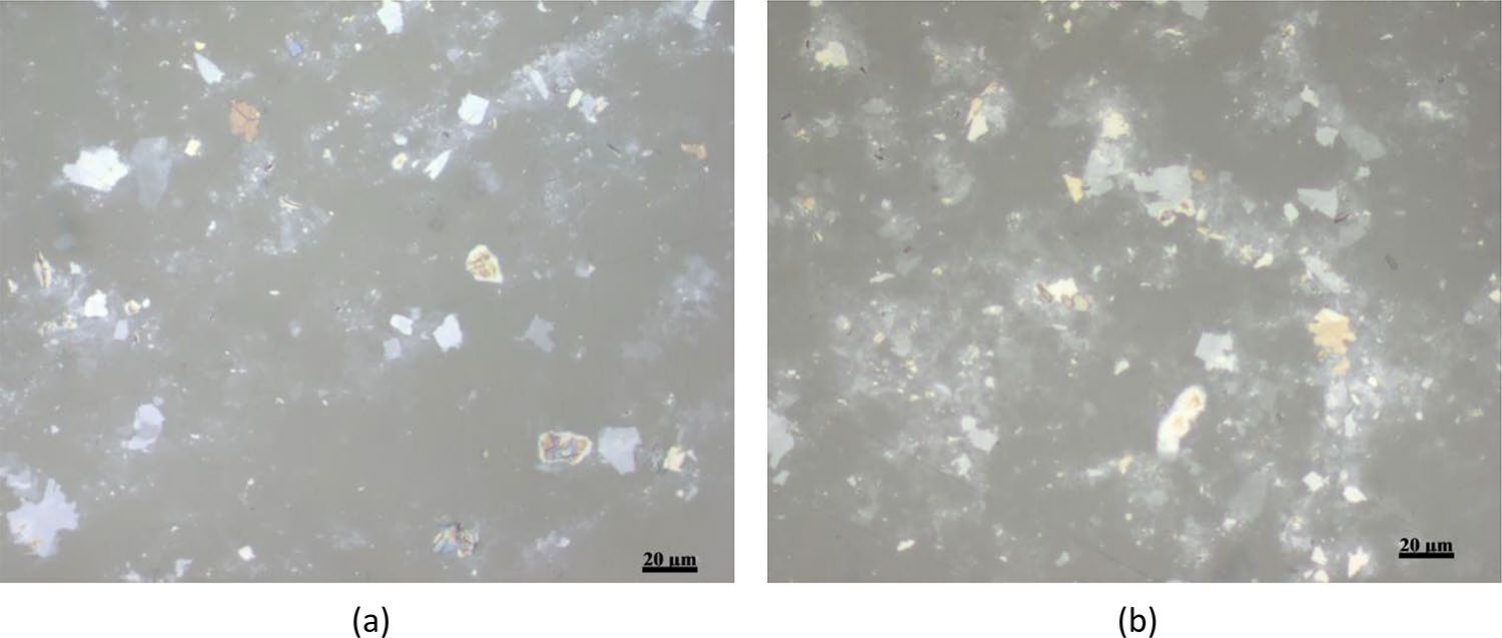
Two microscopic images for the GO film on top of COC wafer **(a)** before and **(b)** after the water boiling test.

It is important to study the stability of the deposited GO film on the COC wafer. To achieve this, JIS K 6744 boiling water test is used for a COC wafer coated with GO. The wafer is left in boiling water for an hour. Microscopic images of the wafer surface are taken before and after the boiling test. As presented in Fig. 4, the distribution of the flakes is not affected which reflects stable GO layers as no peeling off occurs for the deposited film.

## Results and discussion

### RRAM-based tunable filter

**Figure 5:**
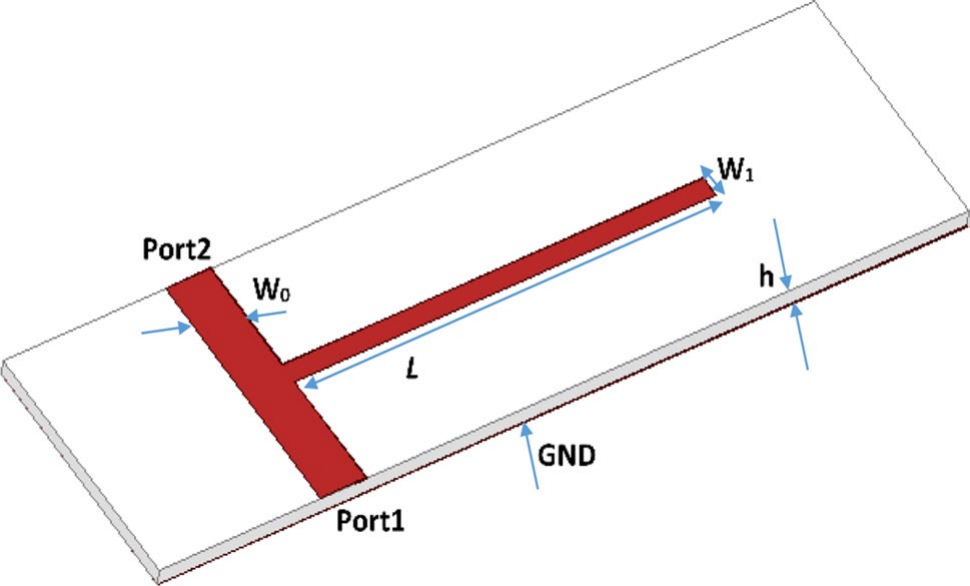
Structure of the open stub notch filter.

The parametric simulation study of the RRAM-based filters is explained in this section. Ansys HFSS is used as simulation software. A standard open stub notch filter design is adopted here^37^. As presented in Fig. 5, the filter is designed on COC substrate (ε_r_ = 2.35, tanδ = 0.0001, substrate thickness = 1 mm). A quarter wave open stub with length *L* is placed at the middle of the main transmission line with width *W*_*0*_. The impedance of the filter is determined by the width *W*_*0*_ which is designed for the standard 50 Ω impedance. The frequency of operation is determined as follows

1 $${\text{F}}\left( L \right) = {c \mathord{\left/ {\vphantom {c {\left( {4*\left( {\varepsilon _{{reff}} } \right)^{{1/2}} L} \right)}}} \right. \kern-\nulldelimiterspace} {\left( {4*\left( {\varepsilon _{{reff}} } \right)^{{1/2}} L} \right)}}$$

where *c* is the speed of light, *ε*_*reff*_ is the effective permittivity of the substrate and *L* is the length of the open stub.

**Figure 6:**
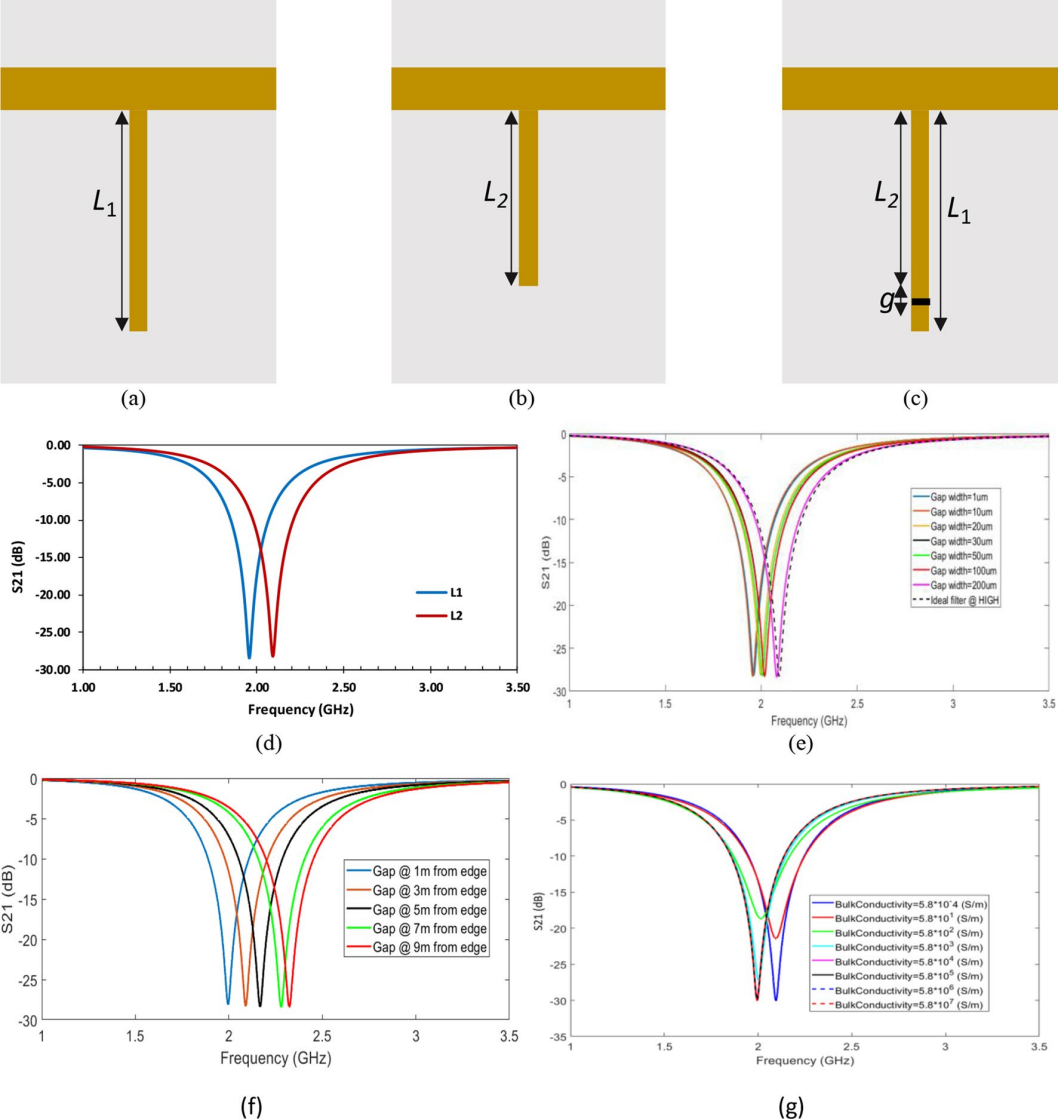
The Structure and frequency characteristics of the open stub notch filter **(a)** Structure of ideal filter with full length. **(b)** Structure of ideal filter with short length. **(c)** Structure of RRAM-based filter. **(d)** S21 parameters of filters presented in **(a, b)** with *L*_1_ = 26.46 mm and *L*_2_ = 24.46 mm. The effect of gap with fixed *L*_2_ **(e)** size, **(f)** position, and **(g)** conductivity on the filter characteristics.

The main aim of this study is to explore the filter characteristics with RRAM device integrated within its metal. This has critical role in guiding the tunable filter design and fabrication. The switching ability of RRAM can provide tuning feature through changing the resonating length of the open stub according to the resistance state of the device. To achieve clear understanding about this concept, two filters with different lengths (Fig. 6a, b) are simulated and their transmission characteristics are compared to each other. As shown in Fig. 6d, shorter filter length results in higher resonance frequency, which is in agreement with Eq. (1).

To demonstrate the tuning concept proposed in this work, a resistive gap element is modeled and integrated within the open stub as presented in Fig. 6c. The resistive gap is supposed to be in the HIGH resistance state or the LOW resistance state depending on the last conductance value written on it. If the RRAM switch is in HIGH state, the filter will choose only the shortest resonating length (*L*_2_) that pushes the resonance frequency to a higher level. While when the switch is at LOW state the filter will choose the resonating length as the total length (*L*_1_) leading the cut-off frequency to shift to a lower frequency level.

The gap width size (*g*) is an essential parameter to be studied and understood. The reason is that even if the gap has HIGH resistance, its size should be big enough to eliminate any coupling that can take place via the gap^36^. To clarify this, the transmission characteristic of the filter presented in Fig. 6c is simulated with different gap size (*g*) that varies from 1 to 200 µm. The conductivity of the gap switching material is set as 5.8 × 10^–4^ S/m to simulate the HIGH state. The S-parameters for the different gap sizes are provided in Fig. 6e and compared to the ideal characteristic that corresponds to the filter shown in Fig. 6b with length *L*_2_. It can be observed that the filter with the integrated resistive gap can have ideal behavior if the gap size is ≥ 200 µm. However, it is challenging to operate a RRAM device with such gap (the distance between the two electrodes) size as the generated electric field will not be sufficient to switch the state of the device, taking into account the planar structure of the device^41–44^. The gap of RRAM devices reported in the literature ranges from nm to submicrons^39^. From Fig. 6e, the gap size of 20 µm has been chosen for RRAM-based filters as it is practical to operate RRAM device with this gap size and at the same time it gives remarkable shift in the frequency. To achieve higher shift in the resonance frequency of the tunable filter, the position of the gap can be shifted towards the main transmission line. The simulation results of this study are revealed in Fig. 6f.

The aforementioned studies are carried out to set a framework to design the size and the position of the resistive gap to be integrated within the filter. Another important parameter to be explored is the conductivity of RRAM gap. At a fixed gap size and position, the conductivity of the gap switching material defines the characteristics of the filter. As presented in Fig. 6g, it is found that in ‘HIGH’ resistive state (when the resistance of the gap is too high, of the order of mega ohms), the filter has better rejection. On the same way, at ‘LOW’ resistance state (when the resistance of the memristor is low, of the order of few kilo ohms), the filter has better rejection. For all other values of resistances, the filter rejects moderately.

### Planar GO-based resistive switching

**Figure 7:**
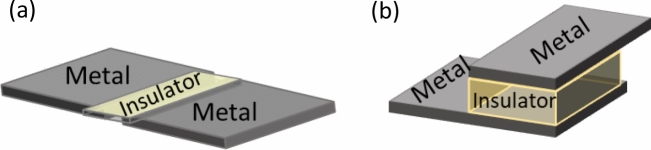
**(a)** Planar and **(b)** stacked RRAM structures.

**Figure 8:**
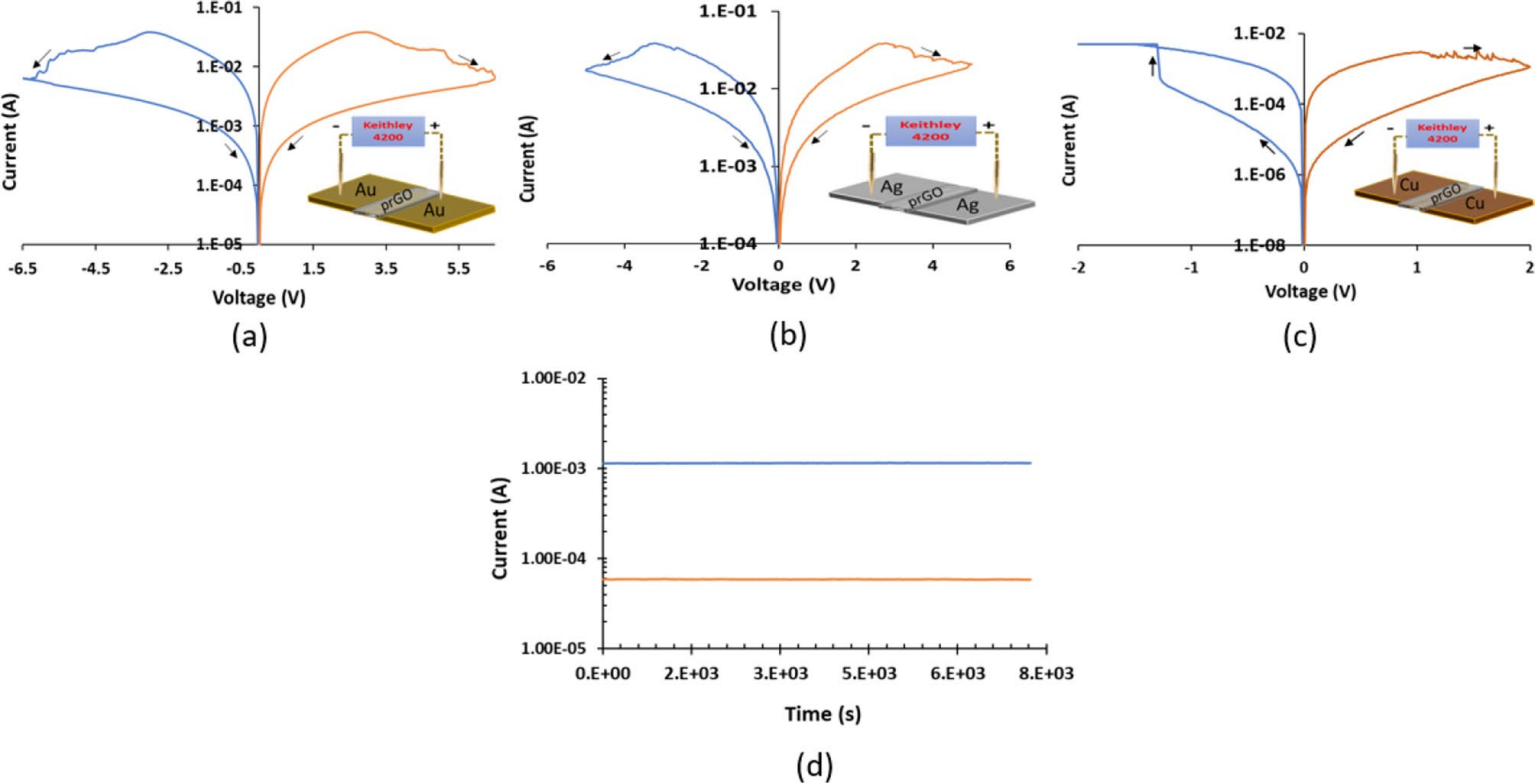
I–V characteristics of resistive switching devices. Unipolar switching behaviour exhibited using **(a)** Au electrodes. **(b)** Ag electrodes. **(c)** Bipolar switching behaviour exhibited using Cu electrodes. **(d)** Chart illustrating the retention of RRAM device presented in **(a)** for ON and OFF states under the application of DC reading voltage of 0.1 V for 2 h.

Graphene based switching devices are gaining great interest due to their excellent properties in terms of low cost, flexibility, adaptability, and being environmentally friendly^34,45–50^. It has been shown that deploying graphene as electrodes in RRAM devices can increase its conductivity and thus improve the device performance. Additionally, using GO as the switching layer in RRAM can assist in multi-resistance switching behavior which is considered an asset in many emerging applications. From RRAM device point of view, it is challenging to operate the device in planar structure (Fig. 7a), rather than the stacked structure (Fig. 7b) that is well reported in the literature^39^. Switching the active material in RRAM device (Metal/Insulator/Metal structure) requires high electric field to be generated, which can be easily achieved in the stacked structure. For more clarification, generating high electric field can be accomplished by diminishing the distance between the metal electrodes (in nm range) while providing suitable active area (between the electrodes) that facilitates building and rupturing the conductive filaments. In the filter tuning concept proposed in this work, the resistive gap should be deployed in a planner manner and as proved in Fig. 6, the gap should be ≥ 20 µm to eliminate any coupling that can take place via the gap during OFF state. Thus, design an RRAM device with the dimentions and the materials that can be integrated in a tunable RF filter is the main challenge in this work. In this section three planar GO-based resistive switching devices fabricated by our group are presented and explained. Three different metals for the electrodes are used; Au, Ag and Cu. The devices are fabricated in planar structure to facilitate its integration within the filter metal. Figure 8 shows the electrical characteristic of each device. It can be observed that Au and Ag systems (Fig. 8a,b) exhibit unipolar switching behaviour. In such characteristic, the switching behaviour of the device does not depend on the polarity of the applied voltage; thus, the same I–V curve can be obtained in either polarity. On the other hand, the Cu system shown in Fig. 8c presents bipolar switching, where the device switching direction depends on the applied voltage polarity^39^.

**Figure 9:**
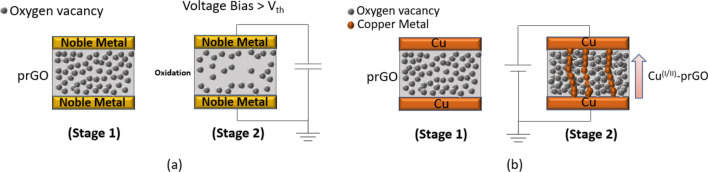
I–V Switching mechanism associated to GO-based switching device fabricated with **(a)** noble electrodes (i.e. Au, Ag), **(b)** Cu electrodes.

The mechanisms associated to both switching behaviours are provided in Fig. 9. For Au and Ag systems, as the used electrodes are noble metals (Fig. 9a), the switching in the oxide layer happens due to the oxidation taking place in the partially reduced GO film (prGO), without any contribution from the metal electrodes ^39,51,52^. This occurs upon the application of sufficient voltage (V ≥ V_th_) across the device, where the threshold voltage V_th_ depends on the gap between the metal electrodes (20 µm is used here). The synergistic effect of the applied voltage V_th_ and the current passing through the device generates Joule heating that causes oxidation of the oxygen vacancies and consequently changes the resistance of the switching film. To confirm that Joule heating is the dominant force for the system presented in Fig. 9a, the device is tested under vacuum (i.e. pressure = 5 × 10^–5^ Pa) and it shows to preserve its switching behaviour. Creating and rupturing of conducting filaments via Joule heating-based reduction and oxidation is called fuse-antifuse and it is well reported in the literature of RRAM switching mechanisms^39^. As for the Cu system presented in Fig. 9b, when positive voltage is applied across the device, copper cations are dissolved from the active electrode and conductive filaments are built towards the positively charged electrode. This mechanism is called electrometallization (ECM) and it is associated to active electrodes deployed in resistive switching devices^12,53^.

It is worth mentioning that the three resistive switching systems presented in Fig. 8 have the potential to be deployed in tunable RF filter, depending on the target application of the communication element. In this work, as a proof of concept, Au system is utilized to demonstrate the proposed RRAM-based filter tuning. As shown in Fig. 8d, a detailed retention test has been performed at room temperature for 2-h for both ON and OFF state and the RRAM device proved its stability and ability to hold its last written state. In addition, multiple devices have been tested for retention and confirmed retention for over 1-year.

### Tunable filter measurements

**Figure 10:**
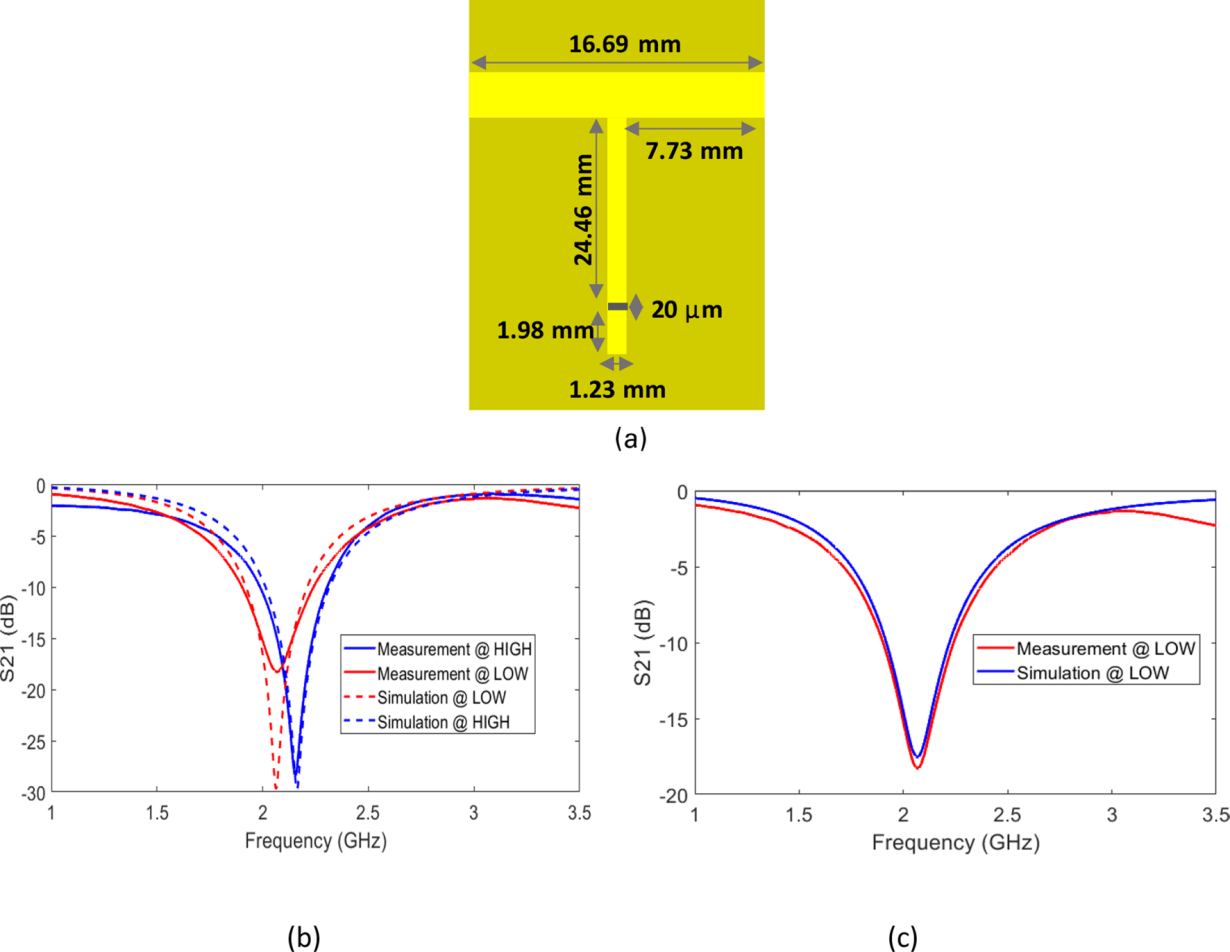
Measurement results of the RRAM-based fabricated notch filter using gold. **(a)** Structure and dimensions of the filter. **(b)** S21 parameter for the measurement and simulation results for both LOW and HIGH states. **(c)** S21 parameter for the measurement and simulation results for LOW state after tuning the simulation ON resistance value of the resistive gap to match the measurements.

The framework detailed in the previous sections is followed to guide the fabrication of RRAM-based notch filter using gold metal. Gold has been used due to its high conductivity and excellent properties for RF applications^38^. The GO material is integrated within the filter to provide the tuning property. The filter schematic and measurements are shown in Fig. 10. The HIGH (ON) and LOW (OFF) states insertion losses of the filter are plotted in Fig. 10b. A frequency tuning range of 85 MHz is obtained. Comparing the filter measurements to the simulation results provided on the same figure, the dB value is correct for the HIGH state which is − 28 dB in simulation and the same for measurement. This indicates the good matching with the used 50 W feed line and the connector. The LOW state has less dB in measurement (− 18 dB in measurement and − 28 dB in simulation). The difference in the loss between the filter simulation and measurement is due to the ON resistance of the resistive gap (100 Ω) which is lower than the resistance value used in the simulation. This resistance level is obtained due to the use of 0.01 A compliance current during switching the gap resistance. In general, a compliance current is used during the characterization of RRAM devices to protect the device from the high current that can affect its functionality and switching ability^39^. The fabricated band stop filter exhibits a suppression of about − 18 dB at the centre frequency, which corresponds to about 0.016 as power attenuation factor, which is still good for real-world applications. The mismatch in terms of in-band suppression with the simulation is due to modelling the RRAM ON conductance with a value equal to the metal conductance 5.8 × 10^7^ S/m. Thus, by updating the simulation parameters and use the ON resistance value obtained in the measurements, this diminishes the mismatch between the measurements and simulations (Fig. 10c).

Table 1 displays the switching performance of the fabricated RRAM-based tunable filter compared to existing works. It is clear that this work is the only experimental one compared to RRAM-based tunable RF components available in the literature. As shown by the experimental results, the proposed concept to tune the center frequency of microstrip notch filters by employing a resistive switching device works perfectly. This idea can be implemented with other filter types, taking into account the framework provided to simulate and design the target RRAM-based filter.

**Table 1:** Performance comparison of proposed RRAM-based tunable filter with other works.

Category	This work	^19^	^20^	^21^	^26^	^27^	^29^	^30^
Design type	BSF	Antenna	Loop filter for PLL	Phase shifter	BPF	BPF	Antenna	BPF
Frequency (GHz)	2	2.308 and 3.143	1	10^–8^ and 10^–6^	2.44 and 5.3	1.6 and 3.5	2.88–4.62	10
Minimum ON state resistance (Ω)	100	0.5	100	316,000	3.6	2.1	2.5	3
Maximum OFF state resistance (Ω)	1 × 10^6^	2000	16,000	632,000	3.6 × 10^12^	2.1 × 10^12^	–	–
Switching device	RRAM	RRAM	RRAM	RRAM	RRAM	RRAM	PIN diode	PIN diode
Device prototype	Experiments	Simulations	Simulation	Simulations	Simulations	Simulations	Experiments	Experiments

## Conclusions

Tunable filters are essential elements as their frequencies of the passband or rejection band can be varied by adjusting their components or parameters. The paper proposed utilizing the resistance switching ability of RRAM devices to tune the behavior of communication filters. This is achieved by integrating the switching device in the filter design. This, then allows varying the effective lengths in the filter by applying sufficient voltage to tune the resistance of the RRAM device. The paper provided detailed simulation analysis for standard notch filter with the concept of tuning the filter using resistive switching material. The proposed idea was demonstrated experimentally by fabricating notch filter using gold as conductive material. The results exhibited by the fabricated filter showed promising insights that is considered milestone in the research of RRAM-based adaptable communication systems.
